# Methylation clocks for evaluation of anti-aging interventions

**DOI:** 10.18632/aging.206245

**Published:** 2025-05-05

**Authors:** Josh Mitteldorf

**Affiliations:** 1 Independent Researcher, Philadelphia, PA 19119, USA

**Keywords:** methylation, stochastic, entropy, programmed aging, aging clock, epigenetic clock

## Abstract

Methylation clocks have found their way into the community of aging research as a way to test anti-aging interventions without having to wait for mortality statistics. But methylation is a primary means of epigenetic control, and presumably has evolved under strong selection. Hence, if methylation patterns change consistently at late ages it must mean one of two things. Either (1) the body is evolved to destroy itself (with inflammation, autoimmunity, etc.), and the observed methylation changes are a means to this end; or (2) the body detects accumulated damage, and is ramping up repair mechanisms in a campaign to rescue itself. My thesis herein is that both Type 1 and Type 2 changes are occurring, but that only Type 1 changes are useful in constructing methylation clocks to evaluate anti-aging interventions. This is because a therapy that sets back Type 1 changes to an earlier age state has stopped the body from destroying itself; but a therapy that sets back Type 2 changes has stopped the body from repairing itself. Thus, a major challenge before the community of epigenetic clock developers is to distinguish Type 2 from Type 1. The existence of Type 1 epigenetic changes is in conflict with conventional Darwinian thinking, and this has prompted some researchers to explore the possibility that Type 1 changes might be a form of stochastic epigenetic drift. I argue herein that what seems like directed epigenetic change really is directed epigenetic change. Of five recent articles on “stochastic methylation clocks,” only one (from the Conboy lab) is based on truly stochastic changes. Using the Conboy methodology and a methylation database, I construct a measure of true methylation drift, and show that its correlation with age is too low to be useful.

## INTRODUCTION

Methylation-based aging clocks have been in use since 2013. One uniquely important application is for evaluating anti-aging interventions. Human trials are long and costly. Methylation clocks promise a quick and inexpensive way to determine if a given intervention has power to rejuvenate, and a slower but reasonably convenient way to measure effects on the rate of aging.

We might assume, naively, that there is something called “epigenetic age”, and that it always predicts a person’s mortality and future life expectancy. Then we might use “epigenetic age” as a marker to determine whether a given intervention is associated with rejuvenation. This paradigm is often implicit, but it is untenable, given what we know about diverse modes of aging [1–3].

Recent literature on the subject has acknowledged two forms of epigenetic changes that occur with age: programmed and stochastic. Stochastic changes vary from one individual to the next and may be due to random variation in the biochemical environment that has no evolutionary purpose. By “programmed changes” I shall mean changes in gene expression that are the result of natural selection; they are presumed to be adaptive, though the adaptive benefit may be to a community or population, not necessarily to the individual. I will argue herein that within programmed changes, some are actually detrimental to the individual, while others are beneficial to the individual, and I will refer to these as Type 1 and Type 2, respectively. Teasing them apart is difficult. But it is essential to distinguish them, because we want our interventions to affect these in opposite directions relative to their age-dependent direction.

N.B. It is well-known and acknowledged that some CpG sites typically gain methylation with age and others lose methylation with age. The distinction between Type 1 and Type 2 is separate from and orthogonal to hypomethylation vs. hypermethylation.

## Theories of aging

There is wide agreement that some methylation changes with age are stochastic and others are programmed. The stochastic changes are likely to have detrimental effects on the body; but what of the programmed changes?

The biology community is divided over the question whether aging is, in part, a programmed phenomenon. Evolutionary theory that has been foundational since the 1920s tells us that aging can only lower individual fitness, defined as reproductive potential or the Malthusian parameter. The idea that aging could be affirmatively selected in a Darwinian process is a non-starter. Hence, there are several well-regarded alternative explanations [4] for the way that aging came to be such a common feature of metazoic organisms.

I have argued from phenomenology and ecology that aging is programmed [5]. The genetic machinery that regulates lifespan has been conserved for at least a billion years – a strong indication that it must have an adaptive purpose [6, 7]. This conclusion is reinforced by the finding that there is low additive genetic variance for aging [8, 9]. The fact that animals routinely increase their lifespan in response to starvation and other hardships is called *hormesis***.** The caloric restriction effect would not be possible unless lifespan is maintained at a sub-optimal level when plenty of food is available [10, 11]. The fact that lifespan is so readily extended in model organisms by disabling a gene that is prevalent in the wild type also suggests that natural selection is not trying to maximize lifespan (even subject to constraints) [12, 13]. I have taken up the challenge to the evolutionary theory that these findings pose, and proposed an evolutionary mechanism based on demographic stability [5, 14].

### Suppose there is no pro-aging epigenetic program

For those who believe that aging is purely an accumulation of damage, with no programmed component, how are they to regard directed methylation changes that are clearly not stochastic? They must conclude that methylation changes are a response to damage. The body perceives that it is in deepening trouble, and repair mechanisms are ramped up with age. I refer to these changes as Type 2.

Suppose a methylation clock is built on Type 2 changes. Then, according to the clock, “younger” means “less repair activity.” “Older” means “better protected”. An intervention that sets back the methylation age, as measured by Type 2, is deceiving us — the intervention nominally lowers “epigenetic age”, but it is likely to actually decrease life expectancy.

This is not a hypothetical situation, outside of general experience. For example, the Horvath Grim Age clock [15], is one of the most popular and most accurate for predicting mortality and time-to-death. The largest component of CpG sites in the GrimAge clock is derived, statistically, from a difference between smokers and non-smokers. We can ask, why should smokers and non-smokers have different methylation profiles? It is a reasonable conjecture that smokers’ bodies are constantly trying to repair their lungs. Much of the methylation signature of smoking consists in up-regulated repair mechanisms. But in the GrimAge clock, smoking is counted as a pro-aging factor, because smokers have curtailed life expectancy. Suppose we are studying an intervention, and we find that it sets back the GrimAge clock. It is possible that this is because the body has promptly repaired lung damage, and so the body has decided that the urgency of repair is reduced and has down-regulated repair mechanisms in response. In this case, the GrimAge clock has correctly identified an anti-aging effect. But it is more likely that the intervention has directly down-regulated the repair mechanisms, leaving the damage unaffected. Then our intervention will score (by GrimAge) as an anti-aging success, but the score will be deceptive. The intervention has only dialed down repair, and it is likely it will shorten life expectancy.

### Suppose there is a pro-aging epigenetic program

For those who believe there is a programmed aspect to aging, we expect that epigenetic changes that progress with age may be a form of programmed self-destruction. For example, inflammation may be dialed up; repair modalities may be dialed down; or apoptosis may be excessive. I call these Type 1 epigenetic changes. An intervention that sets back epigenetic age according to a purely Type 1 clock has decreased the body’s self-destruction, and its effect on life expectancy is likely to be beneficial. This methylation clock works as we expect it to.

But biologists who believe in programmed aging also recognize that there are Type 2 changes with age. The body is at war with itself, and as time goes on, the self-destruction overtakes the repair. But right up to the end, the body is detecting damage, and those repair mechanisms are increasingly activated.

All statistical methodologies for identifying methylation trends associated with aging mix Type 1 and Type 2 indiscriminately. But “younger” according to Type 1 is a good thing, and “younger” according to Type 2 is a bad thing.

For those who believe there is a programmed aspect to aging, it becomes imperative to distinguish Type 1 from Type 2. We wish to construct a clock that contains only Type 1 methylation sites. Better still, we might wish for a clock based on Type 1 minus Type 2 — the Type 2 changes should be negatively weighted in the clock, as compared to their age-associated direction.

For those who believe that there is no programmed component to aging, things are simpler. Of course, there are stochastic methylation changes that take place over time, and these are likely to be detrimental to the individual. But stochastic changes are, by definition, problematic for constructing an aging clock, because there is no consistency from one individual to another. Concerning methylation changes that take place consistently with age, they cannot be causes of decline (according to our assumption), so (to the extent that they are consistent across the population) they must be responses to the damage that accumulates with age. These are Type 2 changes that invoke repair mechanisms. Therefore, methylation clocks should be read in reverse! If there is no programmed component to aging, any intervention that sets back methylation age is killing us, where an intervention that increases methylation age is extending our life expectancy. Caveat: There may be some interventions that work so rapidly and effectively that the damage is repaired, and the body responds by dialing down repair mechanisms, and this is read appropriately as reduction in epigenetic age.

Conversely, if there are known life extension interventions that push methylation markers toward a lower epigenetic age, this fact is prima facie evidence that Type 1 changes exist, and, therefore, that there is a programmed component to aging.

## Stochastic clocks

This collision between the technology of epigenetic clocks and the Neo-Darwinism evolutionary paradigm has percolated for a decade, but only erupted into the academic literature in 2024. The proposed resolution has been to regard detrimental changes in gene expression that appear late in life as random events, attributed to degradation of information.

There are manifest problems with this interpretation. Whether a given methylation change is directed or stochastic is sometimes difficult to determine, and authors with different perspectives are able to project their prejudices into this ambiguity. My presumption is that methylation changes over a lifetime are directed unless proven otherwise. My prejudice in this regard derives from the fact that, of necessity, gene expression is tightly regulated in general. Proper gene expression in different cells at different times is essential to a functioning organism.

- If a CpG site is unmethylated (β=0) at the start of life, and becomes progressively methylated with age, this may be modeled as a random walk constrained at one endpoint, but, by the reasoning above, it is more likely to be adaptive.
- Similarly, if a site is methylated at the start of life (β=1), there is nowhere for β to go but down, and again, I would presume that its time evolution is directed unless proven otherwise.
- If a CpG site is partially methylated at the start of life (0.2<β<0.8) and β changes in a consistent direction through the lifespan, this is *prima facie* evidence that the change is directed.
- If a CpG site is partially methylated at the start of life (0.2<β<0.8) and β changes in a direction that is almost equally likely to be positive in some individuals and negative in others, this is *prima facie* evidence that the change is stochastic.

In the analysis that follows, I will use the terms “stochastic change” or “epigenetic drift” to mean exclusively those changes that can be presumed stochastic because the pattern varies widely from person to person, or because, averaging over individuals, the lifetime change is small compared to the scatter. CpG sites that begin life too close to β=0 or β=1 can drift in only one direction; therefore, I regard such sites as indeterminate as to whether epigenetic change over a lifetime is drift or programmed. This is a different standard from that which has been adopted in 4 of the studies reviewed herein.

The Conboy Lab [16] published a broadside against extant methylation clocks and included at the end a description of methodology for creating a methylation clock based on stochastic drift. They choose sites for which β changes negligibly with age (on average), and construct their clock from those individual deviations from average β which tend to grow with age. They do not report the results of this experiment. I have tried a similar methodology, reported below.

Meyer and Schumacher [17] use simulated (computer-generated) methylation data to demonstrate how one would go about constructing a methylation clock based not on directed methylation changes but on loss of methylation information. The authors proceed to build a clock around DNA sites that begin life either 100% methylated or unmethylated. In either case, random change can only occur in one direction. In these cases, it is difficult to distinguish random change from directed change, as I argue above. They claim that “accumulating stochastic variation in purely simulated data is sufficient to build aging clocks,” with the caveat that “our simulations may not explicitly rule out a programmed aging process”. My interpretation is that it cannot be determined whether the methylation changes on which their (biology-free) clock is based are programmed or stochastic.

Tarkhov et al. [18] is premised on the explicit assumption that aging is not programmed, and that any changes in methylation with age are either random drift or a response to damage (what I have called Type 2). They cite Hayflick [19] as a source for the idea that aging has an origin in the Second Law of Thermodynamics, a paradigm that was discredited for good reason in the 19th Century. Living systems are open systems that take in free energy and dump their entropy into the environment. There is no physical necessity for entropy to increase. This is underscored by the fact that there are some animals and many plants that do not age, in the sense that their mortality risk declines for long segments of the life history [20, 21]. Some animals are able to revert, when starved, to a larval stage, and begin life anew [20]. Ironically, it was Hayflick’s signature discovery [22] that forms the basis of the earliest documented measure of aging at the cellular level, which has certainly evolved and is not entropic.

Tarkhov introduces the concept of “co-regulated” methylation, defined as CpG islands that become fully methylated or fully unmethylated as a unit. They regard every partially methylated CpG island as stochastic change. They ignore the possibility that there may be functional logic to a partially methylated CpG island, perhaps allowing partial expression of an adjacent gene. Our understanding of the role of methylation in epigenetic regulation is still sketchy, but for those who adhere to the neo-Darwinian perspective, there seems to be a need to frame detrimental epigenetic changes as happenstance rather than adaptation.

Tong et al. [23] ask the question: how much of the computation in the most popular Horvath methylation clocks is attributable to directed methylation changes, and how much of it is stochastic? Their conclusion, based on a computer model, is that most of it is stochastic. However, in order to construct that model

- They start with the sites that have already been chosen by Horvath because they change most consistently with age.
- For each of these, they note what direction the site changes with age, and how much methylation of that site changes with each passing year.
- They feed that information into an algorithm that “randomly” adds or subtracts methylation to each particular site with probability calculated to reproduce the observed rate. The process is “random” only in that the exact timing of each methylation addition or subtraction is random. But the probability has been pre-adjusted so that the average rate will match the measured rate according to the Horvath clock.

When the curtain is pulled back from the assumptions in their model, it is clear that their conclusion (that methylation changes as incorporated in the Horvath clock [24] are stochastic) is not in line with the definition of “stochastic” that I have presented herein.

Of these five studies, Markov et al. [25] is the most conservative. They emphasize the difficulty of separating stochastic lab errors from stochastic drift in epigenetic patterns. But the way they propose to solve this problem is to look only at sites that are either fully methylated or fully unmethylated early in life. I have argued above that “drift” that is limited to a single direction cannot be statistically distinguished from directed change.

## How can truly stochastic methylation changes be identified?

Once we put theoretical expectations aside, it is difficult to separate the stochastic component from the directed component of methylation changes over a lifetime. One clear way to identify changes that are likely to be stochastic was suggested by Mei et al. [16].

- Choose among CpGs that are neither fully methylated nor fully unmethylated early in life.
- Select those CpGs for which there is a larger population variance in β later in older vs. younger populations.

As a pilot study, I attempted to construct a true stochastic methylation clock (as I define it) based on this premise. (Details in Supplementary Materials1, 2.) I used a pre-cleaned database of 278 individuals, age 2 to 92, with methylation data from the Illumina 480K array.

Most sites begin life fully methylated or fully unmethylated; for these, drift can be in only one direction, so drift and directed change look much the same. As a first step to finding sites where stochastic change with age could be identified unambiguously, I eliminated sites with a minimum β<0.2 or a maximum β>0.8. Only 5% of CpGs remained (about 24,000).

I worked with the logistic transform of β, defined as logit(β)=ln(β/(1-β)). (β ranges from 0 to 1, and logit(β) ranges from - ∞ to + ∞. I filtered for CpGs where average β does not change over the lifespan, but variance in β increases. For each site, I calculated average and standard deviation of the logit for the young (<35) and old (>70) population subsets.

There were 587 CpGs for which the stochastic change in β overwhelmed the directed change in β. I defined this to be the case when the increase in the standard deviation in logit(β), young to old, was more than 10x the change in the average of logit(β) (young to old).

For each individual, for each of these 587 CpGs, I calculated the squared difference between the individual’s logit(β) and the population mean logit(β). The sum (over 587 sites) of these squared differences was taken as a measure of how far the individual had drifted. (These are all sites for which the average β is roughly independent of age.)

**Figure 1:**
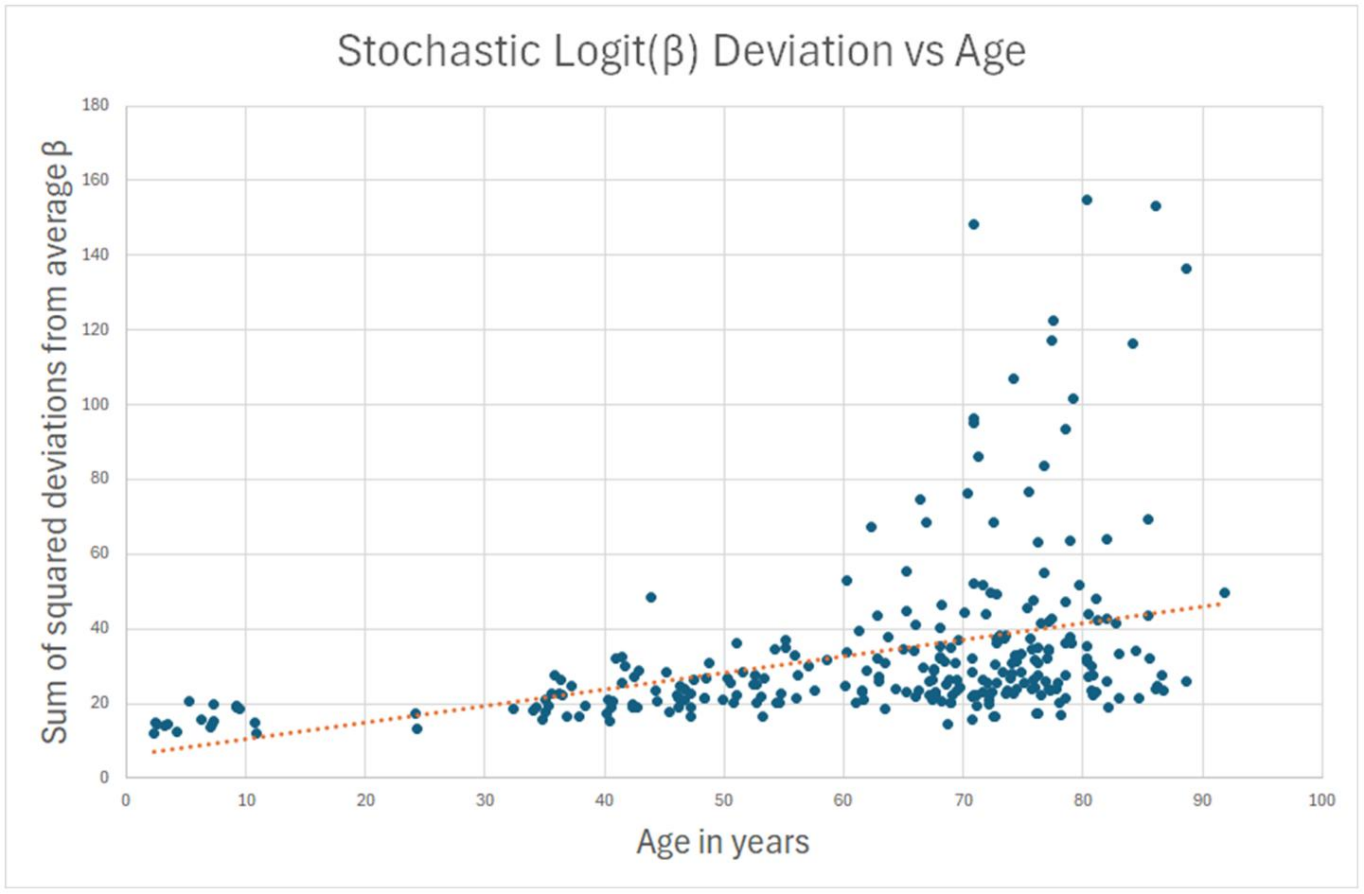
**Sum of squared deviations from beta values that don’t change over a lifetime is tried as an age estimate.** Correlation is only 0.38, and only about 10% of subjects have significantly increased deviations at advanced age.

My intent was to construct a methylation clock derived from truly stochastic change (as I have defined it above) from these 587 sites, using this RMS drift as a criterion. However, I found that its correlation with age was only 0.38. In Figure 1, you can see that in about 25 individuals (10% of the sample), there was a statistically significant methylation drift over a lifetime.

**Figure 2:**
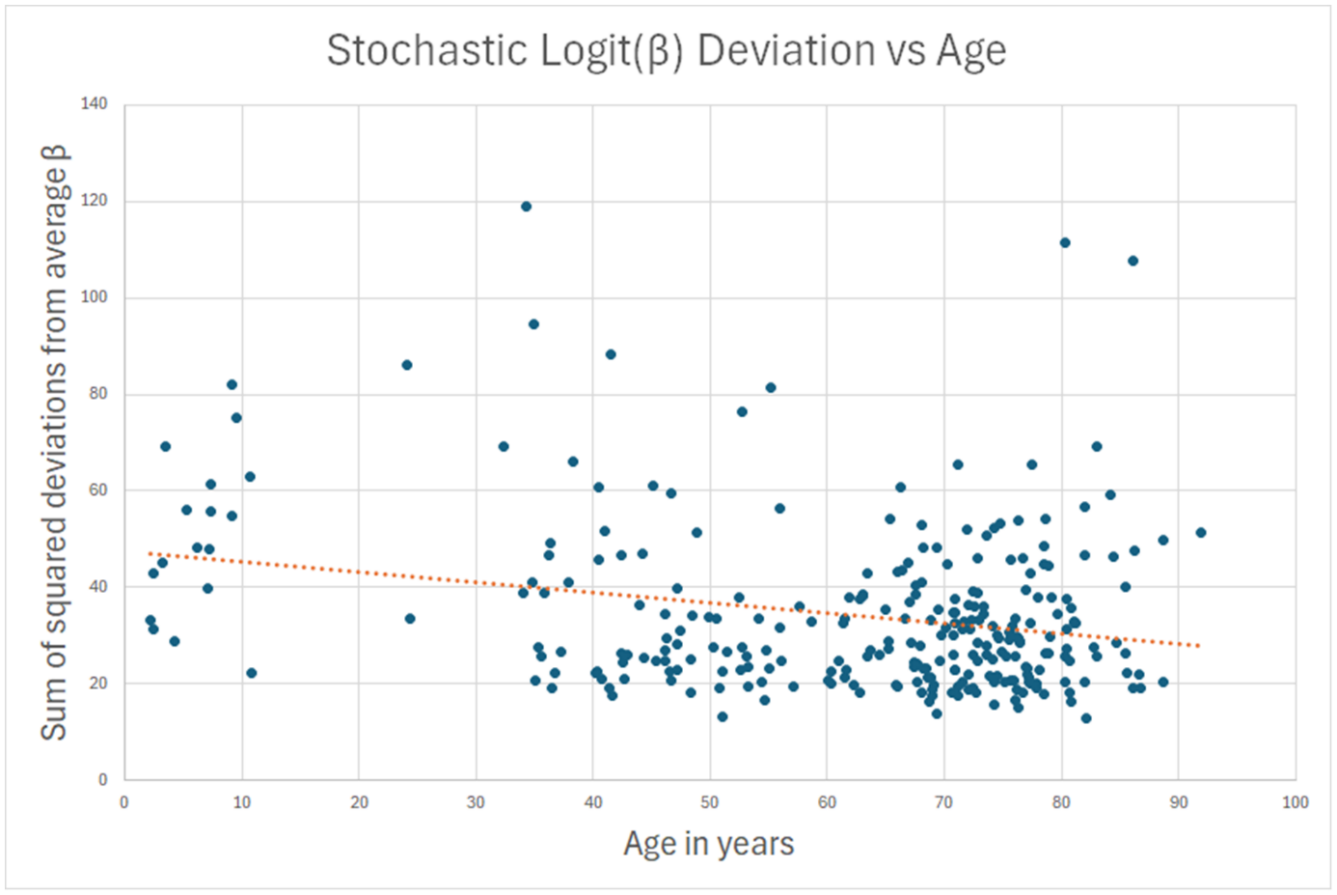
**A sham clock was created using the betas that had less scatter in the oldest subjects than in the youngest.** This data is presumed to be all lab error, and it can be used to estimate lab error in the actual stochastic clock of Figure 1.

My hypothesis was that for the remaining 90%, the measured methylation disparity represents measurement error. To test this hypothesis, I repeated the analysis for sites where standard deviation of methylation *decreased* (young to old) over a lifetime, and the decrease was more than 10x the absolute change in average logit(β). There is no biological or physical reason why methylation should “undrift” over a lifetime, becoming less stochastic with age. Hence, the data in this analysis can safely be assumed to be measurement error. This second reverse-stochastic pseudo-clock (Figure 2) showed scatter comparable to the 90% in the true stochastic clock, and correlation with age of -0.25.

### Tentative conclusions

In this attempt to construct a methylation clock based on purely stochastic drift, I found that in only about 10% of the sample there was sufficient stochastic drift to separate their methylation profiles from background noise.

Of course, this is only a small pilot study; but the result suggests that stochastic drift in methylation is not a promising basis for an epigenetic clock.

## DISCUSSION

The most important use of aging clock algorithms is to evaluate anti-aging interventions in humans without having to wait decades to collect mortality data. Presently, the most accurate and robust measures of biological age [26] are based on patterns of methylation.

But methylation, as a form of epigenetic regulation, is presumptively under tight evolutionary control. If methylation changes with age in a directed way, this can have only two purposes:

1. The body is programmed to turn on self-destructive genetic activity late in life.
2. The body perceives increased damage suffered with age and ramps up repair processes in response.

The possibility that Type 1 changes exist is denied by a majority of aging researchers today, who believe in a classical version of Darwinian selection. In this paradigm, deliberate self-destruction would be antithetical to individual fitness and could never be selected in nature.

For those who embrace this paradigm, this leaves only Type 2 changes. The implication is that methylation changes associated with late life represent the body’s rescue response. They are, therefore, beneficial. Paradoxically, an intervention that “sets back” the body’s methylation clock to a younger state is shutting off vital repair mechanisms, so it is likely inimical to health and longevity. Thus, methylation clocks are useless — or worse — for evaluation of anti-aging interventions.

Methylation clocks have been in use for a decade, but only in the last year, this paradox has evoked a response from the majority paradigm. Five recent research articles explore the idea that most methylation changes with age are not programmed, but constitute random drift. This is a third possibility, distinct from the directed changes of Type 1 and Type 2.

This hypothesis is dubious at its root, because epigenetic regulation is among the most tightly controlled of metabolic processes. Correct epigenetic expression is essential for every aspect of biological function.

All five articles classify some epigenetic changes as (stochastic) drift without establishing a proof that they are not directed under evolutionary control. In particular, if a methylation site begins life fully methylated or fully unmethylated, then “drift” can be in one direction only, and there is no way to distinguish whether it is directed change or, indeed, it is true drift.

I have proposed criteria for identifying methylation changes that are truly stochastic.

- The site should remain partially methylated through the lifetime. I have set boundaries at 0.2 < β < 0.8.
- The average β should not change appreciably across life span.
- Individual, idiopathic deviation from this average β can be identified with confidence as true stochastic drift.

About 5% of methylation sites covered in the Illumina 480K array meet these criteria. My attempt to construct an epigenetic clock based on such sites failed badly, because true drift could only be identified above the noise in a small percentage of the sample population.

I proffer this as evidence in favor of the proposition that most epigenetic change that occurs consistently over the human lifetime is not stochastic, but is directed, of Type 1 or Type 2.

It is not easy to distinguish Type 1 from Type 2, but a clock based on a mixture of Type 1 and Type 2 methylation is likely to produce inconsistent and misleading results, when applied to anti-aging technologies. Therefore, the experimental separation of Type 1 from Type 2 should be a research priority before epigenetic clocks can be useful in their evaluative function.

## Supplementary Material

Supplementary Material 1

Supplementary Material 2
