# Supplementary material for: Methylation clocks for evaluation of anti-aging interventions
Source: Aging (Albany NY). 2025 May 5;17(5):1082–90. doi: 10.18632/aging.206245 (PMC12151506; doi:10.18632/aging.206245)
Supplement: Supplementary Material 1 [file aging-17-206245-s001.docx]

Supplementary Material 1. Delphi Pascal program generating data for stochastic methylation clock.

program StochasticClock;

{$APPTYPE CONSOLE}

{ DEFINE OVER40}

{$J+} {$R+}

(* 1) transform all betas using ln(beta) - ln (1-beta) so the range is - infinity to infinity.

2) calculate correlation with age for each site

3) Sort by correlation, and throw out the top 90% in absolute value

4) Among those bottom 10% correlation with age, divide age into quartiles. Calculate variance for 1st and 4th quartile.

5) List the sites that have the greatest difference between variance for 4th quartile minus variance for 1st quartile. *)

uses SYSUTILS,DOSSTUFF;

{Choose the best 1000 age-related CpGs from an array with many thousands.

Our criterion for a good age-related CpG is

the squared linear regression coefficient vs age divided by

the residual variance of beta after dependence on age is removed.

Update: a little algebra reveales that my criterion function is R^2/(1-R^2) divided by the variance of the age sample.

So selection by R^2, as Horvath has done, should be entirely equivalent. Spreadsheet output confirms that it is equivalent.}

const {nsites=198;}

numeralset=['0'..'9','.','E'];

nchosen=1000;

totalsubj=278;

tab=#9;

type rarray=array[1..totalsubj] of double;

pair=record nn :integer; crit :double; end;

str10=string[10];

var s :string;

fin,fout :text;

fint :file of pair;

age,meth :rarray;

lbeta :array[1..totalsubj,1..500000] of double; {485258}

id :array[1..500000] of str10;

corr2 :array[1..500000] of double;

dummy :array[1..13] of char;

ch :char;

c :array[0..900000] of pair;

nsites :integer;

nvara,suma,

suma2 :double;

h50 :array[1..50] of str10;

index :word;

bestr2 :double;

nsubj :word;

best :array[1..30000] of integer;

procedure ReadHorvathTop50;

var i :byte;

begin

assign(fin,'c:\d\Aging\AClock\DNAMethDataControls\Top50of353.txt'); reset(fin);

for i:=1 to 50 do

readln(fin,h50[i]);

close(fin);

end;

function FoundInHList(target :str10):boolean;

var i :byte;

begin

result:=true;

i:=1;

while (i<50) do

if (target=h50[i]) then exit else inc(i);

result:=false;

end;

procedure CopyHorvath50;

var s,olds :string;

target :str10;

i,j :integer;

begin

assign(fin,'c:\temp\BigData.csv'); reset(fin);

assign(fout,'Horvath50fromBigData.csv'); rewrite(fout);

readln(fin,s); writeln(fout,s);

i:=0; j:=0;

repeat

inc(i);

olds:=s;

readln(fin,s);

{if (FoundInHList(s)) then begin writeln(fout,s); inc(j); end;}

target:=copy(s,2,10);

if (FoundInHList(target)) then begin writeln(fout,s); inc(i); inc(j); end;

(*

writeln(fout,olds,crlf,s);

readln(fin,s); writeln(fout,s); inc(i); inc(j);

end;

*)

if (i mod 1000=0) then write(i:14,':',j:2);

until eof(fin);

close(fin); close(fout);

end;

procedure AddAgesToBigData;

var s :string;

begin

assign(fin,'Horvath50FromBigData.csv'); reset(fin);

readln(fin,s); readln(fin,s);

close(fin);

assign(fin,'c:\temp\BigData.csv'); reset(fin);

assign(fout,'BigData.csv'); rewrite(fout);

writeln(fout,s);

repeat

readln(fin,s);

writeln(fout,s);

until eof(fin);

close(fin);

close(fout);

end;

procedure QSort(l,r: integer); {From Borland demo}

var

i,j :integer;

swap :pair;

x :double;

begin

i:=l; j:=r; x:=c[(l+r) div 2].crit;

repeat

while (c[i].crit>x) do inc(i);

while (x>c[j].crit) do dec(j); {reverse order, largest crit first}

if (i<=j) then begin

swap:=c[j]; c[j]:=c[i]; c[i]:=swap;

inc(i); dec(j);

end;

until (i>j);

if (l<j) then QSort(l,j);

if (i<r) then QSort(i,r);

end;

procedure QSortPos(l,r: word); {From Borland demo}

var

i,j :word;

swap :pair;

x :integer;

begin

i:=l; j:=r; x:=c[(l+r) div 2].nn;

repeat

while (c[i].nn<x) do inc(i);

while (x<c[j].nn) do dec(j); {reverse order, largest crit first}

if (i<=j) then begin

swap:=c[j]; c[j]:=c[i]; c[i]:=swap;

inc(i); dec(j);

end;

until (i>j);

if (l<j) then QSortPos(l,j);

if (i<r) then QSortPos(i,r);

end;

procedure ReSortByFilePos;

begin

QSortPos(1,nsites);

end;

procedure SortPairs;

begin

QSort(1,nsites);

end;

procedure CopyFirst200;

var i :word;

begin

{assign(fin,'c:\d\Aging\AClock\DNAMethDataControls\BigData.csv'); reset(fin);}

{assign(fin,'c:\d\Aging\AClock\BigData21Feb.csv'); reset(fin);}

assign(fin,'c:\temp\BigData.csv'); reset(fin);

assign(fout,'First20.csv'); rewrite(fout);

for i:=1 to 20 do begin

readln(fin,s);

writeln(fout,s);

end;

close(fin);

close(fout);

end;

procedure ReadNumber(var x:double);

var s :string;

ch :char;

ta :integer;

begin

s:='';

repeat read(fin,ch) until (ch in numeralset);

while (ch in numeralset) do begin s:=s+ch; read(fin,ch); end;

val(s,x,ta);

end;

(*

procedure OpenFiles;

var i,j :word;

s :string;

ta :word;

wr :double;

begin

{assign(fin,'First200.tsv'); reset(fin); {"tab-separated var"}

assign(fin,'c:\d\Aging\AClock\DNAMethDataControls\BigData.csv'); reset(fin);

readln(fin,s); for i:=1 to 13 do begin read(fin,ch); write(ch); end; writeln;

for i:=1 to nsubj do begin read(fin,age[i]); writeln(age[i]:12:9); end;

readln(fin);

n:=0;

end;

procedure OpenFiles;

var i,j :word;

ch :char;

s :string;

ta :word;

wr :double;

begin

assign(fin,'c:\d\Aging\AClock\DNAMethDataControls\BigData.csv'); reset(fin);

readln(fin,s); repeat read(fin,ch); write(ch); until (ch=','); writeln;

{$ifdef OVER40} for i:=1 to tooyoung do ReadNumber(age[i]); {$endif}

for i:=1 to nsubj do begin ReadNumber(age[i]); writeln(i:2,age[i]:12:7); end;

readln(fin,s); writeln(s);

nsites:=0;

suma:=0; suma2:=0;

for i:=1 to nsubj do begin suma:=suma+age[i]; suma2:=suma2+sqr(age[i]); end;

nvara:=suma2-sqr(suma)/nsubj;

{halt;}

end;

procedure OneSite;

var i :word;

beta :array[1..nsubj] of double;

sumb,sumb2,sumab, num,denom2,r2,residual,A2 :double;

begin

repeat read(fin,ch) until (ch=',');

{$ifdef OVER40} for i:=1 to tooyoung do ReadNumber(beta[i]); {$endif}

for i:=1 to nsubj do ReadNumber(beta[i]);

readln(fin);

sumb:=0; sumb2:=0; sumab:=0;

for i:=1 to nsubj do begin

sumb:=sumb+beta[i];

sumb2:=sumb2+sqr(beta[i]);

sumab:=sumab+age[i]*beta[i];

end;

num:=sumab-suma*sumb/nsubj;

A2:=sqr(num/nvara); {square of linear coefficient}

residual:=(sumb2-sqr(sumb)/nsubj - A2*nvara)/nsubj;

inc(nsites);

c[nsites].nn:=nsites; c[nsites].crit:=A2*nsubj/residual; {Our criterion for a good age-related CpG is

the squared linear regression coefficient vs age divided by the residual variance of beta after dependence on age is removed.}

if (nsites mod 1000=0) then writeln(nsites:8,c[nsites].crit:10:4);

end;

*)

procedure NumberfromString(s :string; var index :word; var x:double);

var ss :string;

ta :integer;

begin

ss:='';

inc(index);

while s[index] in numeralset do begin

ss:=ss+s[index];

inc(index);

end;

val(ss,x,ta);

end;

procedure OpenFiles;

var i,j :word;

ch :char;

s :string;

ta :word;

wr :double;

begin

nsubj:=278;

assign(fin,'BigData.csv'); reset(fin);

readln(fin,s); index:=4; s:=s+',';

for i:=1 to nsubj do begin NumberfromString(s,index,age[i]); writeln(i:2,age[i]:12:7); end;

readln(fin,s); {writeln(s);}

nsites:=0; bestr2:=0;

suma:=0; suma2:=0;

for i:=1 to totalsubj do {$ifdef Over40} if age[i]>40 then {$endif} begin suma:=suma+age[i]; suma2:=suma2+sqr(age[i]); end;

{$ifdef OVER40} for i:=1 to totalsubj do if (age[i]<=40) then dec(nsubj); {$endif}

nvara:=suma2-sqr(suma)/nsubj;

{readln; halt;}

end;

procedure OneSite;

const crit=0.07; infinity=100.0;

var i :word;

sumb,sumb2,sumab, num,denom2,r2,residual,A2,nvarb :double;

beta :array[1..totalsubj] of double;

CpG :string;

begin

inc(nsites);

readln(fin,s);

CpG:=copy(s,2,10);

id[nsites]:=CpG;

index:=0; repeat inc(index) until (s[index]=',');

s:=s+',';

for i:=1 to totalsubj do begin

NumberfromString(s,index,beta[i]);

if (beta[i]<1E-6) then lbeta[i,nsites]:=-infinity else if (1-beta[i]<1E-6) then lbeta[i,nsites]:=infinity

else lbeta[i,nsites]:=ln(beta[i])-ln(1-beta[i]);

end;

sumb:=0; sumb2:=0; sumab:=0;

for i:=1 to totalsubj do {$ifdef Over40} if (age[i]>40) then {$endif} begin

sumb:=sumb+lbeta[i,nsites];

sumb2:=sumb2+sqr(lbeta[i,nsites]);

sumab:=sumab+age[i]*lbeta[i,nsites];

end;

nvarb:=sumb2-sqr(sumb)/nsubj;

r2:=sumab - suma*sumb/nsubj;

if (nvarb<0.01) then r2:=1.0

else r2:=sqr(r2) / (nvara*nvarb);

corr2[nsites]:=r2;

if (corr2[nsites]>0.5) then dec(nsites);

if (nsites mod 1000=0) then writeln(nsites:8,r2:10:4,bestr2:10:4);

end;

procedure WriteSortFile;

var i :integer;

begin

assign(fint,'SortedPairs,dat'); rewrite(fint);

for i:=1 to nsites do write(fint,c[i]);

close(fint);

end;

procedure ReadSortFile;

begin

assign(fint,'SortedPairs,dat'); reset(fint);

nsites:=0;

repeat

inc(nsites);

read(fint,c[nsites]);

until eof(fint);

close(fint);

write('NSites=',nsites); readln;

end;

(*

procedure OneSite;

var i :word;

suma,summ,sumaa,summm,sumam, num,denom2,r :double;

begin

for i:=1 to 13 do read(fin,ch); {read(fin,dummy);}

for i:=1 to pred(nsubj) do read(fin,meth[i]); readln(fin,meth[nsubj]);

suma:=0; summ:=0; sumaa:=0; summm:=0; sumam:=0;

for i:=1 to nsubj do begin

suma:=suma+age[i];

sumaa:=sumaa+sqr(age[i]);

summ:=summ+meth[i];

summm:=summm+sqr(meth[i]);

sumam:=sumam+age[i]*meth[i];

end;

num:=sumam-suma*summ/nsubj;

denom2:=(sumaa-sqr(suma)/nsubj)*(summm-sqr(summ)/nsubj);

r2[n].rsq:=sqr(num)/denom2; r2[n].nn:=n; {<AM>-<A><M> / sqrt((<AA>-<A><A>)(<MM>-<M><M>))}

writeln(n:8,r2[n].rsq:10:4);

end;

procedure OpenFiles;

var i,j :word;

s :string;

ta :word;

wr :double;

begin

assign(fin,'First200.csv'); reset(fin);

readln(fin,s); for i:=1 to 13 do begin read(fin,ch); write(ch); end; writeln;

for i:=1 to pred(nsubj) do begin

s:='';

repeat

read(fin,ch); s:=s+ch;

until(ch=',');

s:=copy(s,1,length(s)-1);

val(s,wr,ta);

{read(fin,age[i]); read(fin,ch);}

age[i]:=wr;

writeln(age[i]:12:9);

end;

readln(fin,age[nsubj]);

n:=0;

end;

procedure OneSite;

var i :word;

suma,summ,sumaa,summm,sumam, num,denom2,r :double;

begin

for i:=1 to 13 do read(fin,ch); {read(fin,dummy);}

for i:=1 to pred(nsubj) do read(fin,meth[i],ch); readln(fin,meth[nsubj]);

suma:=0; summ:=0; sumaa:=0; summm:=0; sumam:=0;

for i:=1 to nsubj do begin

suma:=suma+age[i];

sumaa:=
